# The two-network framework of number processing: a step towards a better understanding of the neural origins of developmental dyscalculia

**DOI:** 10.1007/s00702-022-02580-8

**Published:** 2023-01-20

**Authors:** Elise Klein, André Knops

**Affiliations:** 1 LaPsyDÉ, UMR CNRS 8240, Université Paris Cité, La Sorbonne, 46 Rue Saint-Jacques, 75005 Paris, France; 2 Leibniz-Institut Fuer Wissensmedien Tuebingen, Tuebingen, Germany

**Keywords:** Learning disorders, Arithmetic fact retrieval, Magnitude processing, Connectivity, Numerical cognition

## Abstract

Developmental dyscalculia is a specific learning disorder that persists over lifetime and can have an enormous impact on personal, health-related, and professional aspects of life. Despite its central importance, the origin both at the cognitive and neural level is not yet well understood. Several classification schemas of dyscalculia have been proposed, sometimes together with an associated deficit at the neural level. However, these explanations are (a) not providing an exhaustive framework that is at levels with the observed complexity of developmental dyscalculia at the behavioral level and (b) are largely mono-causal approaches focusing on gray matter deficits. We suggest that number processing is instead the result of context-dependent interaction of two anatomically largely separate, distributed but overlapping networks that function/cooperate in a closely integrated fashion. The proposed two-network framework (TNF) is the result of a series of studies in adults on the neural correlates underlying magnitude processing and arithmetic fact retrieval, which comprised neurofunctional imaging of various numerical tasks, the application of probabilistic fiber tracking to obtain well-defined connections, and the validation and modification of these results using disconnectome mapping in acute stroke patients. Emerged from data in adults, it represents the endpoint of the acquisition and use of mathematical competencies in adults. Yet, we argue that its main characteristics should already emerge earlier during development. Based on this TNF, we develop a classification schema of phenomenological subtypes and their underlying neural origin that we evaluate against existing propositions and the available empirical data.

## Introduction

There is broad consensus that numerical information is processed in a multimodular and distributed fashion in the human brain. The triple-code model (Dehaene and Cohen 1995, 1997) has been remarkably successful in providing a framework for this distributed processing as it suggested that there are different codes for processing different aspects of numerical information which can be assigned to different brain regions (Dehaene and Cohen 1995, 1997). The most prominent distinction is between an analogue, abstract and automatic magnitude code in a bilateral fronto-parietal network centered around the bilateral intraparietal sulcus (IPS) and frontal structures on the one hand, and a verbal code for the retrieval of highly overlearned arithmetic facts comprising left-hemispheric perisylvian areas and the angular gyrus (AG) on the other. Furthermore, the model proposes a visual code involved in recognizing (strings of) Arabic digits and number words. This visual code is supposed to be located in close vicinity to the visual number form area (VNF) within the fusiform gyrus in the inferior occipito-temporal region (Yeo et al. 2017). Its functional specificity and lateralization remain a matter of debate (Amalric and Dehaene 2016; Grotheer et al. 2018; Yeo et al. 2020). Not only does the TCM posit a close interaction between these codes (Dehaene and Cohen 1997), but the gray matter areas that underlie the individual codes (for meta-analyses, see Arsalidou et al. 2018; Arsalidou and Taylor 2011; Dehaene et al. 2003; Hawes et al. 2019) also need to communicate with each other (Klein et al. 2013). Therefore, we proposed an amendment to the triple-code model in which we added dorsal and ventral white matter fiber tracts that connect the gray matter areas underlying magnitude and arithmetic fact retrieval processing (Klein et al. 2016). This two-network framework (TNF) of numerical cognition and its successors comprises two anatomically largely separate networks for magnitude processing and arithmetic fact retrieval which operate closely together as functionally integrated circuits (Göbel et al. 2022; Kaufmann et al. 2020; Klein et al. 2013, 2016). The interrelation of the two codes was suggested to be reciprocal and to depend on the task demands and difficulty: the more a task requires the manipulation of quantities and the application of procedures, the less fact retrieval processes were supposed to be involved, and vice versa. However, we suggested that a certain (minimal) amount of fact retrieval and magnitude processing is always involved in numerical processing independent of the difficulty of the task. For example, the result of a multiplication problem such as 3 × 8 is supposedly represented in a phonological format that is independent from a numerical semantic elaboration. In parallel, however, we may engage in a rudimentary and approximate magnitude estimation process that allows us to immediately refute proposals that are numerically too far of (e.g., 3 × 8 = 3400). This, in turn, requires magnitude processing to some extent. On the other hand, a more complex task such as 372 + 491, which draws heavily on the manipulation of magnitudes and procedures may be broken down into its constituting components, for example retrieval of arithmetic facts such as 2 + 1 = 3 from long-term memory.

The proposed amendment to the TCM was based on correlative data (fMRI and DTI) from healthy participants with typical numeracy (Klein et al. 2016). Thus, the question arises whether or not the described areas and connections are indeed causally involved in the numerical processes suggested and, thus, whether the two-network framework can sufficiently explain numerical performance of cognitively impaired participants as well. Especially in arithmetic fact retrieval, it has been criticized that several of the areas, which are typically found activated during arithmetic fact retrieval, may neither be specific to nor necessary for arithmetic fact retrieval (e.g., Bloechle et al. 2016; Grabner et al. 2009a, b; Grabner et al. 2009a, b; Ischebeck et al. 2007). The angular gyrus, for instance, has been frequently associated with arithmetic fact retrieval (e.g., Delazer et al. 2003a, b; for a meta-analysis see Dehaene et al. 2003), but was also modulated by learning of mathematical, but also non-mathematical content (Ischebeck et al. 2007). In addition, neuropsychological single-case studies do not unequivocally support a causal role as some patients with a multiplication deficit had a preserved AG (Cohen and Dehaene 2000; Dehaene and Cohen 1997; Zaunmuller et al. 2009), while others with a lesion of the left AG did not show a multiplication deficit (e.g., Van Harskamp and Cipolotti 2001).

Against this background, we recently investigated the role of network disruptions following brain lesions for post-stroke cognitive deficits using disconnectome mapping in acute stroke patients at the group level (Smaczny et al. 2021). This recent method allows to draw inferences on causal associations between lesion-induced disconnections and cognitive deficits as it quantifies the effects of focal brain lesions on the brain’s structural connectome (e.g., Griffis et al. 2019, 2021). The focus of the study was on arithmetic fact retrieval (multiplication facts), but addition and subtraction performance was considered as control (Smaczny et al. 2021). Therefore, the results had the potential to verify or falsify assumptions about the structures proposed in our two-network framework of numerical cognition.

In the following, we will first summarize which model assumptions were validated and which modifications we would further suggest for our extended two-network framework of numerical cognition. In a second step, we scrutinize the question to what extent the two-network framework for numerical processing in adults might be useful for developmental dyscalculia by deducing model-based hypotheses about subtypes and origins of developmental dyscalculia and comparing those against what has been previously proposed.

## A two-network framework of numerical cognition

The *TNF* posits that numerical cognition engages the context-dependent interaction of two distributed and anatomically largely segregated networks (Klein et al. 2016). Based on recent data (Göbel et al. 2022; Kaufmann et al. 2020; Smaczny et al. 2021), the following central assumptions of the framework can be specified.

### Two dissociable networks

Consistent with the framework proposed by Klein et al. (2016), Smaczny and colleagues (2021) found evidence for two dissociable networks for magnitude processing and fact retrieval. There was a clear association of multiplication performance with lesion-disconnections in the left hemisphere comprising, among others, the connectivity of AG, superior/middle temporal gyrus, and supramarginal gyrus with the thalamus. In contrast, these disconnections had no effect on solving addition and subtraction problems where the patients’ performance was close to 100%. This dissociation does not only suggest that the observed left-hemispheric disconnections are indeed specific for multiplication fact retrieval; it is also consistent with the framework’s proposition that arithmetic facts are predominantly represented in the left hemisphere while magnitude processing is represented bilaterally. The patients in the study by Smaczny and colleagues (2021) had a first-time unilateral lesion only (either left or right hemisphere). Thus, according to the framework, they should not have presented with a magnitude processing deficit because an impairment of magnitude processing should have indeed required a bilateral lesion. At this point, however, it is important to note that this nevertheless does not exclude that there might be a specialization of the left or right IPS for processing certain numerical content (e.g., non-symbolic quantity processing in the right IPS). The patient data by Smaczny and colleagues (2021) was based on the Number Processing and Calculation Battery (NPC; Delazer et al. 2003a, b) which exploits accuracy data (percentage of correct responses). A deficit (e.g., in subtraction) would only be assigned if the patient either gave an incorrect response or did not respond within 10 s. This leaves ample time for a transfer to the contralateral IPS and compensate a unilateral deficit.

Note that the TNF assumes that the dominant processing type in an arithmetic task (i.e., arithmetic fact retrieval vs. magnitude processing) is related to the difficulty and familiarity of the problem (e.g., simple addition/multiplication vs. difficult addition/multiplication) rather than the operation per se (addition vs. multiplication). Thus, it assumes that arithmetic fact retrieval might not only used in the case of multiplication, but also in the case of other simple and highly overlearned operations such as simple addition. This is in line with studies showing that more difficult (complex) multiplication is associated with the magnitude processing network (e.g., Bloechle et al. 2016; Delazer et al. 2003a, b), with studies showing similar deficit-lesion associations for simple multiplication, addition and even subtraction fact retrieval within the dorsal fiber pathway of the arithmetic fact retrieval network (Mihulowicz et al. 2014), and with studies showing that inhibitory TMS over the AG (i.e., an important fact retrieval area) was detrimental not only for simple multiplication facts but also for simple subtraction facts, while more difficult multiplication (as more difficult subtraction) was not affected (Fresnoza et al. 2020).

Moreover, according to the TNF, arithmetic fact retrieval is also involved in more complex arithmetic as a subprocess in addition to magnitude processing. For instance, the studies underlying our extension of the TCM to the TNF (Klein et al. 2013; 2016) also include re-analyses of different paradigms on complex two-digit addition with approximate/exact computation (Klein et al. 2016) and complex addition with and without carry (Klein et al. 2010). Though exclusively two-digit addition problems were presented, arithmetic fact retrieval was involved as well, depending on the item properties (Klein et al. 2013; 2016). Participants may arguably break down complex problems into more tractable and more familiar pieces allowing for assisting magnitude-related processing by fact retrieval-related components.

### Lateralization

Based on the literature, the two-network framework stipulates that the neural substrate mediating arithmetic fact retrieval is predominantly left-lateralized (Klein et al. 2016). However, the correlative nature of the underlying neurofunctional data did not allow to test for causal implications of the network. The recent findings by Smaczny and colleagues (2021) filled this gap and revealed significant associations between lesion-disconnections and a multiplication deficit only in patients with a left-hemispheric stroke. Multiplication was spared in patients with a right hemispheric damage. Therefore, these data corroborate the notion that arithmetic fact retrieval relies on a predominantly left-lateralized network.

### Widespread network of fact retrieval

**Fig. 1:**
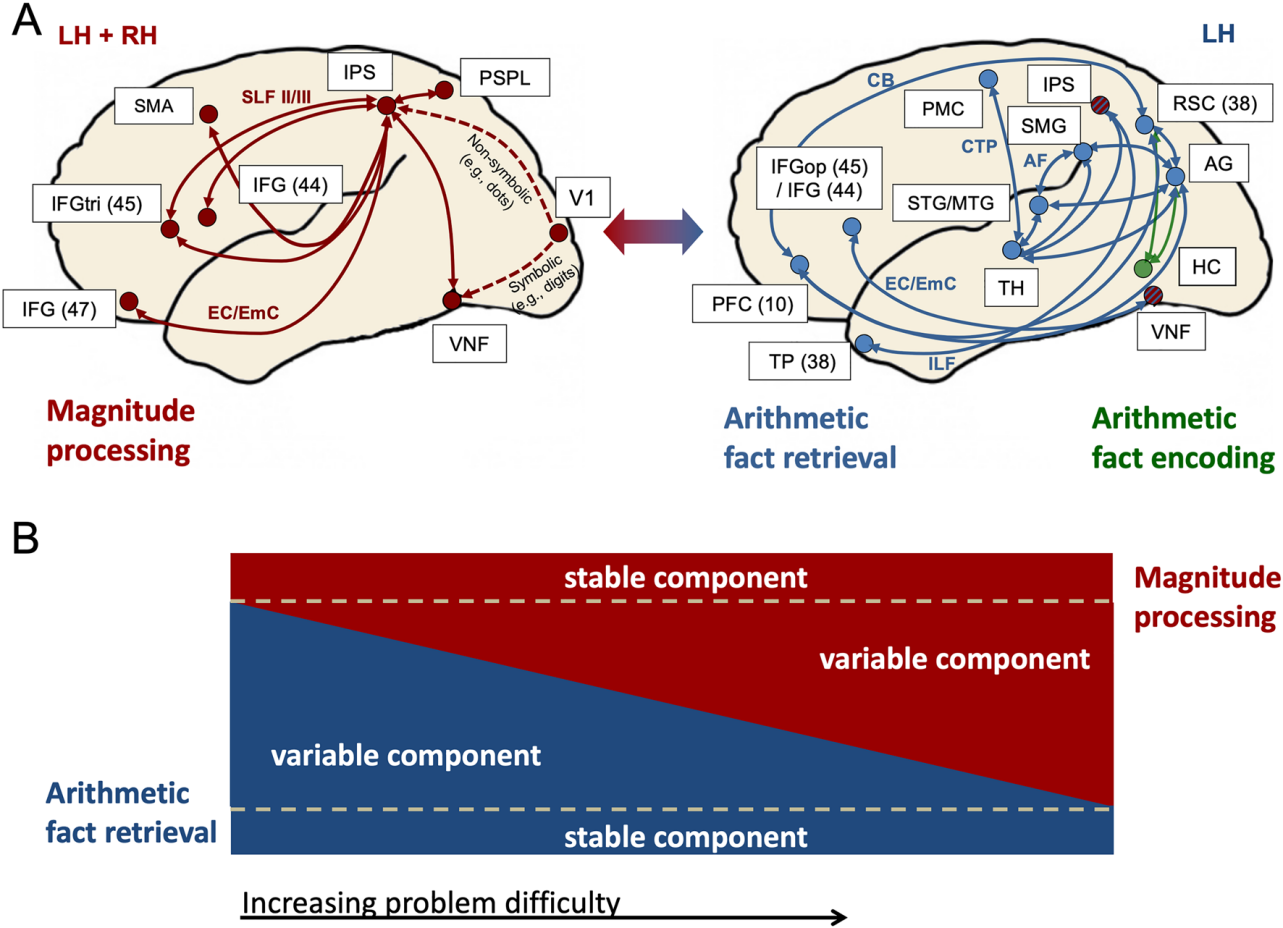
Two-network framework (TNF) of numerical cognition. **A** Cortical networks and processing pathways for magnitude processing (left panel, red) and arithmetic fact retrieval (right panel, dark blue). The color-changing arrow between the two panels reflects that these two anatomically separate, distributed and overlapping networks operate together context dependent as functionally integrated circuits in numerical cognition. During the acquisition of arithmetic facts, the network is supplemented by the hippocampus and entorhinal cortex. **B** Interplay between magnitude processing and arithmetic fact retrieval. A certain amount of fact retrieval (blue rectangle at the bottom) and magnitude processing (blue rectangle at the top) is assumed to be involved in arithmetic independent of the task demands. The more difficult a task, the less fact retrieval (blue triangle, variable fact retrieval component) and the more magnitude processing is involved (red triangle, variable magnitude processing component). *AF* arcuate fascicle, *AG* angular gyrus, *CB* callosal bundle, *CTP* corticothalamic projection, *EC/EmC* external/extreme capsule system, *HC* hippocampus, *IFG* inferior frontal gyrus, *ILF* inferior longitudinal fascicle, *IPS* intraparietal sulcus, *LH* left hemisphere, *op* opercular part, *PFC* prefrontal gyrus, *PMC* primary motor cortex, *PSPL* posterior superior parietal lobule, *RSC* retrosplenial cortex, *RH* right hemisphere, *SLF* superior longitudinal fascicle, *SMA* supplementary motor area, *SMG* supramarginal gyrus, *STG/MTG* superior/middle temporal gyrus, *TH* thalamus, *TP* temporal lobe, *tri* triangular part, *V1* primary visual cortex, *VNF* visual number form area

The TNF originally proposed a relatively small left-hemispheric network of AG, middle temporal gyrus, retrosplenial cortex, hippocampus and prefrontal cortex to underlie arithmetic fact retrieval (Klein et al. 2016). The causal involvement of the AG in arithmetic fact retrieval remained a matter of debate (e.g., Bloechle et al. 2016). Therefore, it is of significance that the recent patient study (Smaczny et al. 2021) indeed confirmed the necessary contribution of the AG in arithmetic fact retrieval. In particular, the whole-brain analysis showed that a disruption of AG connectivity (especially to language areas such as superior/middle temporal gyrus) leads to arithmetic fact retrieval impairment. These findings fit well with the two-network model put forward by Klein and colleagues (2016), as it proposed connections between the middle temporal gyrus and the AG underlying arithmetic fact retrieval (see Fig. 1). However, we suggest to extend the TNF of Klein and colleagues (2016) by the recently identified disconnection clusters associated with arithmetic fact retrieval involving the left arcuate fasciculus, the temporopontine tract and U-fibers between the AG and superior/middle temporal gyrus (Smaczny et al. 2021). This extension and specification of the TNF would explain the seemingly inconsistent findings from previous studies (e.g., Van Harskamp and Cipolotti 2001; Zaunmuller et al. 2009) as it indicates that not only lesions of the AG itself but also various disconnections of the AG and lesions in the vicinity of the AG have a detrimental effect on arithmetic fact retrieval. Additional region-of-interest (ROI) analyses revealed that arithmetic fact retrieval is subserved by a widespread left-hemispheric network including, among others, also temporal areas (superior temporal gyrus, temporal pole), parietal areas (AG, supramarginal gyrus, IPS), frontal areas (inferior frontal gyrus, prefrontal cortex), thalamus, retrosplenial cortex, and the left primary motoric hand area (Smaczny et al. 2021), thereby further extending the network suggested by Klein and colleagues (2016). Taken together, a disconnection of the AG (especially from language areas) leads to fact retrieval impairment. However, not only the gray and white matter integrity of the AG seems to be crucial but also a widespread left-hemispheric network: there are various connections between only two areas, where a lesion in just this connection also leads to an impairment in arithmetic fact retrieval.

It has to be noted that within the widespread fact retrieval network, the AG, supramarginal gyrus, superior temporal gyrus and thalamus seem to be particularly important as they are connected with each other via mono- and polysynaptic connections. These structures can be considered hubs between ventral and dorsal processing pathways as they are not only connected ventrally (e.g., superior temporal gyrus with AG), but also dorsally via the arcuate fascicle.

### Hippocampus

We suggest that the TNF needs to be specified regarding the involvement of the hippocampus and the retrosplenial cortex in arithmetic fact processing. The retrosplenial cortex, which has been found to subserve the recognition of familiarity (see Vann et al. 2009 for a review) and landmarks in spatial mapping (Alexander and Nitz 2015), seems to closely communicate with the hippocampus and angular gyrus during the encoding of arithmetic facts and the retrieval of arithmetic facts (Fig. 1). This was recently corroborated by DTI data showing a significant increase of structural connectivity in fibers encompassing the left hippocampus after multiplication fact drill training (Klein et al. 2019) and by developmental data showing a transient increase of hippocampal activation and functional connectivity during the acquisition of mathematical knowledge (Qin et al. 2014; Rosenberg-Lee et al. 2018). However, once the facts are well consolidated in memory, the hippocampus does not seem to be longer part of the fact network (Smaczny et al. 2021). This is in line with recent data from Delazer et al. (2019) who suggested that hippocampal long-term memory areas together with language areas play a dynamic and time-limited role in encoding and consolidating arithmetic facts (see also Bloechle et al. 2016; Menon 2016), but not in their actual retrieval after consolidation in memory.

### Visual number form area

The earlier versions of the triple-code model (Dehaene 1992; Dehaene and Cohen 1995, 1997) stipulated that the VNF situated in inferior temporo-occipital cortex hosts an asemantic, visual representation of number symbols (visual code). This is in contrast to both the original version of the TNF, which classified the VNF as one of many areas constituting the magnitude processing network (Klein et al. 2016) and the current revised version of the TNF, which lists the VNF as part of both the magnitude processing network and the fact retrieval network (cf. Fig. 1). Thus, in the version of the TNF proposed here, the VNF is considered to subserve visual symbolic numerical processing that provides input to both networks.

Whether the VNF is only a component of the two networks or whether it nevertheless should be considered an independent visual representation is beyond the scope of the current article; however, we wish to note that the processing of numerical magnitude and arithmetic facts is also possible without an involvement of the VNF, e.g., in blind participants (e.g., Dormal et al. 2016), in non-visual stimulus presentation (e.g., auditory; Klein et al. 2010), or in processing of non-symbolic stimuli (e.g., dot patterns; Bloechle et al. 2016). Hence, numerical and arithmetic content can be processed within these two networks independently of VNF. Therefore, we understand the VNF as an area that is optionally recruited whenever the task at hand requires visual symbolic processing.

### IPS and inferior frontal gyrus

Smaczny and colleagues (2021) reported arithmetic fact retrieval deficits following a disconnection of the thalamus not only with the VNF, but also with IPS and the inferior frontal gyrus (IFG Area 44). These three gray matter regions are both part of the arithmetic fact retrieval network and the magnitude processing network. That is, the anatomically largely separate networks seem to overlap in these regions. Based on the existing data, we cannot conclude whether this overlap actually reflects joint recruitment of identical processes or a mere overlap of activation that is due to distinct processes that would be reflected by different processing patterns (e.g., Mock et al. 2019). A central assumption of the TNF is that both networks need to closely interact in a given task context. That requires both networks to communicate, so they actually need to overlap at some gray or white matter region. On a mere speculative level, the IPS (similarly to the VNF for visually presented input, see above) might be a candidate area for such a connection.

In particular, the IPS is widely agreed to be involved in magnitude processing (for reviews and meta-analyses see Arsalidou et al. 2018; Arsalidou and Taylor 2011; Dehaene et al. 2003; Hawes et al. 2019; Knops 2017). The TNF posits that arithmetic tasks are typically not solved exclusively by either arithmetic fact retrieval or magnitude manipulations (i.e., calculations) but by an adaptive interplay between both (Klein et al. 2016). Therefore, we suggest that arithmetic fact retrieval deficits following a disconnection between thalamus and IPS might reflect difficulties in switching between fact retrieval and magnitude manipulation strategies and that the IPS is, therefore, part of both, the magnitude and the fact retrieval network (Fig. 1). In the context of multiplication verification, for example, it has been shown that participants have more difficulties rejecting incorrect but related results (e.g., 4 × 8 = 28) when they are smaller than the correct outcome compared to those who are larger (e.g., 4 × 8 = 36; Didino et al. 2015) despite their equal numerical distance on a linear scale. This has been interpreted as reflecting the logarithmic compression of the mental magnitude representation and, therefore, implies a functional implication of the magnitude system during arithmetic fact retrieval. As a general conclusion, the connection between thalamus and IPS seems to be central for relaying information between the two networks, and receiving, coordinating and transmitting information. Crucially, this means that the TNF assumes that the IPS, although being part in both networks, would subserve magnitude processing resp. the switch to magnitude processing within arithmetic fact retrieval as well.

In addition, the TNF states that both networks should interact in a certain (i.e., reciprocal) fashion. The coordination of retrieval and more elaborate strategies involving the magnitude system may be mediated by the IFG as the prefrontal cortex has been frequently assumed to be involved in strategy selection (e.g., Dehaene et al. 2003).

Taken together, current data do not allow to disentangle whether the overlapping gray matter areas in the two networks represent converging nodes of shared joint processes between networks, mere overlap of functionally distinct processes, or strategy selection. Further studies are needed to substantiate this speculation.

### Assumptions of the TNF

Based on the considerations outlined above, the following falsifiable assumptions can be derived from the TNF:

1. The magnitude representation is located bilaterally in the human brain, i.e., redundantly. Thus, a unilateral lesion of the parietal cortex including the IPS should not result in the inability to compare both symbolic and non-symbolic magnitudes. There might be decreases in response times observed because the content might need to be transferred into the contralateral hemisphere; however, in terms of accuracy data, no deficit should be observed.
2. In individuals with language areas lateralized to the left hemisphere, a unilateral lesion of the contralateral right hemisphere should not lead to a deficit in arithmetic fact retrieval.
3. Although both networks interact closely, it should not be possible to fully compensate a deficit in one network with processes conducted in the other (at least not in terms of completely unaffected response latencies). The TNF proposes that, independent from the difficulty of a task, a small but stable component of processing from the other network is involved and required in all tasks. For instance, individuals who exhibit particular difficulties in arithmetic fact retrieval (e.g., patients suffering from an infarction of the left middle cerebral artery) should not suddenly turn unaffected when a task becomes sufficiently difficult. This clearly not seems to be the case as revealed by a variety of single-case studies.

## Implications for developmental dyscalculia

Most frameworks for numerical cognition consider one fronto-parietal network for number processing that also contains certain specific components for fact retrieval (e.g., Dehaene et al. 2003; Menon 2016) or whose components also process (to varying degrees) arithmetic facts (e.g., Amalric and Dehaene 2016, 2019). In contrast, the TNF posits that there are two segregated, distributed and overlapping networks with dorsal and ventral processing pathways that closely interact—dependent on the context (Klein et al. 2016). The framework is based on healthy adult data and has been able to explain the arithmetic performance of acalculic patients as well (Smaczny et al. 2021) and a few individual cases of dyscalculia in adulthood (Göbel et al. 2022; Kaufmann et al. 2011a, b; for a review, see Kaufmann et al. 2020). However, if the TNF represents the endpoint of the acquisition and use of mathematical competencies in adults, one can assume that main characteristics of the TNF emerge earlier during development. Given this assumed developmental consistency, it should be possible to derive hypotheses about phenotypes of developmental dyscalculia and their underlying ‘neurotype’, i.e., the neural affected neural systems.

Developmental dyscalculia (DD) is a specific learning difficulty in acquiring mathematical skills during childhood which persists into adulthood (for reviews see Knops 2020; Kucian and von Aster 2015; Siemann and Petermann 2018; Von Aster and Shalev 2007). DD is a heterogeneous syndrome that is characterized by multiple cognitive deficits and is associated with various brain dysfunctions. The cardinal deficits relate to difficulties with execution of calculation procedures and arithmetic fact retrieval (Andersson 2010; Landerl et al. 2017; Russell and Ginsburg 1984). In the literature, various etiological views and classifications for subtypes of dyscalculia have been proposed. An influential proposal assumes a dysfunctional magnitude processing within the IPS as the central deficit of dyscalculia, making the IPS as the number sense module of the triple-code model the first dysfunctional instance even before formal learning of arithmetic (e.g., Siemann and Petermann 2018; Von Aster and Shalev 2007). A number of alternative views have been proposed (Butterworth 2005; Geary 2004; Noel and Rousselle 2011; Piazza et al. 2010; Rousselle and Noël 2007) which all share the characteristic of being mono-causal model of dyscalculia. These theories stand in surprising contrast to the variety of cognitive deficits at the phenomenological level. Together with the variety of classification approaches, this suggests that we are still far from fully understanding the etiology of DD—or even the symptomatology of DD. In addition, some classifications neglect the dynamic character of mathematics learning and were based on data from samples that would not allow to even detect certain deficit types. For example, Chan and Wong (2020) outlined several subtypes for DD, but failed to identify a subgroup with a pure arithmetic fact deficit since they only tested 1st and 2nd graders who have not yet had the opportunity to build and consolidate arithmetic fact knowledge.

Starting from the endpoint of mathematical skill development, the TNF suggests four major subtypes of DD, due to either (1) impairments in the fact retrieval network, (2) impairments in the magnitude network, (3) impairments in both networks, and (4) impairments of the interaction of the two networks with each other. For sake of brevity, we will not consider impairments that are not related to any specific aspect of the two networks such as the execution function subtype (e.g., Chan and Wong 2020; Landerl et al. 2017).

### Impairments of/within the fact retrieval network

The cardinal symptom of impairments within the fact retrieval network is the failure to establish stable representations of arithmetic facts (e.g., multiplication tables) in long-term memory. This is indeed the most common impairment observed in DD, which also persists into adulthood (e.g., Landerl et al. 2017). Arithmetic fact retrieval impairments can be identified in several proposed frameworks in the literature (e.g., “arithmetic fact dyscalculia”). Arithmetic fact retrieval impairments have been observed in children with intact (non-symbolic) number processing skills and normal or near-to-normal calculation ability (De Visscher and Noël, 2013; Skagerlund and Träff 2016). Thus, this disorder type may in rare cases not even be diagnosed as dyscalculia, because some diagnostic systems (e.g., DSM-V), only consider the overall performance across all arithmetic operations. Despite the agreement on the principal impairment, different etiologies have been proposed: the hyper-sensitivity to interference hypothesis (De Visscher and Noël, 2013; not specific to calc), the access deficit hypothesis (Rousselle and Noël 2007; see also Skagerlund and Träff 2016) the long-term memory hypothesis (Geary 1993). In the following, we scrutinize each of these propositions against the TNF and the predictions that can be derived from it.

#### Hypersensitivity to interference hypothesis

De Visscher and Noël (2013, 2014) propose that arithmetic fact dyscalculia results from an increased sensitivity to interference. According to this idea, arithmetic facts can be understood as memory items with several constituting features. For example, a problem such as 3 × 9 = 27 comprises the features 3, 9, 2 and 7 (the digits). However, these features overlap with features in previously learned problems such as 3 × 7 = 21 (they share three digits: 3, 7 and 2). The proactive interference from features in previously learned problems on problems that are acquired later during development is supposed to be problematic for children suffering from a hyper-sensitivity to interference and creates difficulties in establishing arithmetic fact representations. According to the authors, increased interference sensitivity is neither due to a general memory deficit nor a general inhibition problem. The persisting interference sensitivity leads to deficits in the consolidation of arithmetic fact learning. Hence, children suffering from interference sensitivity continue to adopt calculation strategies that rely to a large degree on the magnitude network (De Smedt et al. 2011). As to the neural etiology of this problem, the authors suggest two possibilities: either the “hippocampus did not succeed in encoding these associations between problems and answers” (De Visscher and Noël, 2013, p. 67) or impairments in the inferior frontal gyrus might possibly have a detrimental effect on the learning phase of intertwined associations of arithmetical facts. Against the background of the two-network framework, the first problem would concern the connections between hippocampus and angular gyrus and/or hippocampus and retrosplenial cortex (Fig. 1). In both cases, this would imply difficulties in non-mathematical context due to the domain-general involvement of these brain structures in learning and declarative semantic memory. Indeed, De Visscher and Noël (2014) describe a positive correlation between interference sensitivity in numerical and non-numerical contexts (i.e., a non-numerical associative memory task). The overall strength of this correlation of 0.506 which explains only ~ 26% of the overall variance, however, leaves room for alternative or additional underlying deficits in the arithmetic fact network. Hence, we would like to extent the list of possible structures that might underlie this phenotype. Based on the TNF, we hypothesize that some children with arithmetic fact impairments exhibit a structural and/or functional impairment of the long association connections between frontal and prefrontal cortex and (a) angular gyrus, (b) hippocampus or (c) the visual number form area. A plethora of patient studies suggest that impairments of the connection between prefrontal areas and hippocampus lead to deficits in top-down (cognitive or strategic) control of memory processing (e.g., Eichenbaum 2017; Preston and Eichenbaum 2013). These patients were notably impaired in contexts that require the flexible update of previously learned associations such as re-learning a connection of item A with C while A had previously been associated with item B. This deficit is reminiscent of the hyper-sensitivity to interference as stipulated by De Visscher and Noël.

A pattern of structural or functional impairment between frontal areas and visual number form area (see c) above) implies concurrent deficits in recognizing digits and associating semantic meaning, which has been referred to as “access deficit”.

#### Access deficit hypothesis

Skagerlund and Träff (2016) propose that children with arithmetic facts dyscalculia have an access deficit as described by Rousselle and Noël (2007). This hypothesis states that DD in children is not related to problems with numerosity processing (i.e., estimate the approximate numerical magnitude of sets of objects) per se, but rather to problems in accessing magnitude information from symbols (i.e., numerals). These authors argue that fact dyscalculia should be attributable to the impaired connection between symbolic numbers and innate magnitude representations (Rousselle and Noël, 2007; see also Wilson and Dehaene 2007). Therefore, children with arithmetic fact dyscalculia should display problems with symbolic (numerals) number magnitude processing tasks, but perform within normal range on non-symbolic tasks, because only the former type of task requires access to the underlying magnitude representations of Arabic numerals (Rousselle and Noël, 2007; see also Wilson and Dehaene 2007). According to Skagerlund and Träff (2016), four different mechanisms might explain this finding: (a) reduced gray matter volume in the IPS and frontal areas (as observed in Kaufmann et al. 2011a, b), (b) reduced white matter tracts connecting IPS and frontal areas (Nieder 2009), (c) a deficit within the parietal circuit connecting the IPS and angular gyrus (Ansari 2008; Price and Ansari 2011) or (d) an impaired functional connectivity between the hippocampus and angular gyrus (De Visscher and Noël 2013).

Based on the two-network framework, reduced gray matter in frontal areas and IPS (option a) in the above list) should affect strongest the magnitude network, as these constitute its key regions (Fig. 1). The arithmetic fact network might be affected as well, but certainly less prominently. It has to be noted that connections between inferior frontal gyrus and IPS (option b) in the above list) are found in the magnitude network only. Furthermore, according to the TNF, these two brain regions are actually redundantly connected via a dual loop system of dorsal (via superior longitudinal fascicle II) and ventral (via extreme/external capsule system) fiber pathways. This means that observing reduced structural connectivity between IFG and IPS would require a concurrent impairment of dorsal and ventral processing pathways, which should be extremely rare. Finally, from a developmental perspective, it should be noted that these two pathways are not even myelinized during the same time window during development, with the dorsal pathways developing only around the seventh year of age (Friederici 2012). Therefore, we would reject the first two explanations. However, the hypothesis of a deficit in the parietal circuit connecting the IPS and angular gyrus also appears to be inconclusive within the TNF. While an impairment of the U-fibers between AG and IPS may lead to relatively specific impairments in task that require intimate interaction between magnitude processing and fact retrieval, this should be evident predominantly in prolonged reaction time but not prevent the built-up of arithmetic facts, because IPS and AG are polysynapticly connected via the thalamus as well (Fig. 1, see also below). Finally, Skagerlund and Träff (2016) propose that impaired functional connectivity between the hippocampus and angular gyrus might underlie arithmetic fact dyscalculia, because this would impair the memory formation of arithmetic facts. This is not backed by the two-network model, because such a deficit between hippocampus and AG, two structures serving various non-mathematical cognitive processes, would necessarily result in additional difficulties beyond mathematical learning (e.g., when learning to write). In line with this, De Visscher and Noël (2014) found that sensitivity to interference in the numerical tasks was positively correlated with sensitivity to interference in a non-numerical associative memory task in children with low arithmetic fluency. Therefore, we reject the hypothesis by Skagerlund and Träff (2016) that impaired connectivity between hippocampus and angular gyrus can cause specific arithmetic fact dyscalculia.

In the context of the TNF, we would rather expect that a deficit in the connectivity of the left visual number form area (e.g., with frontal areas) would explain arithmetic fact dyscalculia (see also (Rousselle and Noël, 2007). In this case, magnitude processing and calculation (e.g., subtraction) might continue to function largely undisturbed, as it was observed by De Visscher and Noël (2013). However, both impairments (i.e., gray matter or connectivity) would nevertheless have a negative effect on the association between numerical symbols and their numerical magnitude and cause problems with written arithmetic facts. Poorer comprehension, in turn, would hinder the acquisition and consolidation of arithmetic facts in memory. Due to the vicinity of the VNF and the left-lateralized visual word form area, we would hypothesize that children suffering from an access deficit due to an impairment of the connectivity between VNF and AG would be prone to deficits in processing other written symbols, too. This might explain the higher than expected comorbidity between dyscalculia and dyslexia (Moll et al. 2019; Thapar et al. 2017).

#### Long-term memory deficit hypothesis

Geary (Geary 1993; Geary and Hoard 2001) proposed that a long-term memory deficit might underlie DD with impairments in arithmetic fact retrieval. In addition, indeed, a gray matter deficit in the hippocampus or the parahippocampus would be in accordance with problems in semantic memory representations which might lead to an arithmetic fact deficit (e.g., Rykhlevskaia et al. 2009). However, since the hippocampus is involved in learning and memory consolidation beyond the arithmetic or mathematical domain, this impairment should not lead to a circumscribed arithmetic fact retrieval deficit with otherwise spared memory formation (Landerl et al. 2017). Rather, it should manifest more in terms of a generalized learning disorder.

Based on the TNF, we would like to propose that the left-hemispheric regions comprising AG, superior/middle temporal gyrus and supramarginal gyrus, which are inter-connected with mono- and polysynaptic fibers (Fig. 1), might form a module which is central for the acquisition of arithmetic facts together with the hippocampus. These areas constitute a subnetwork, which may subserve the acquisition, encoding and retrieval of arithmetic facts. Disconnections of this subnetwork have been shown to underlie selective arithmetic fact retrieval impairments (Smaczny et al. 2021). Even more importantly, it was recently shown that increased covariance of structural and functional connectivity within this subnetwork is associated with DD (Michels et al. 2022).

### Impairments in the magnitude network

The magnitude network comprises nodes in parietal, frontal and (temporo-) occipital cortex and their fiber connections. These are largely embedded within a superordinate fronto-parietal task-positive network (Fox et al. 2005), which performs various further cognitive functions, many of which are unrelated to number processing. Therefore, in case of impairments within this superordinate network, both number-specific and domain-general processing can be disturbed. Thus, the DD subtypes that affect the magnitude network are divided into number-specific and number-unspecific subtypes.

#### Number-specific subtypes

Skagerlund and Träff (2016) contrast Arithmetic fact dyscalculia (see above) with general dyscalculia. In general dyscalculia, the access to the approximate number system (ANS) or the ANS itself is supposed to be defective. This leads to an impairment of both non-symbolic and symbolic number processing, because the preverbal ability to represent quantities approximately in the ANS is supposed to constitute the foundation for development of the symbolic number system used for arithmetic (e.g., Butterworth 1999; Piazza 2010). Depending on the author, this mono-causal deficit is called defective ANS hypothesis (Skagerlund and Träff 2016), core deficit hypothesis (Wilson and Dehaene 2007), or the number sense deficit (Chan and Wong 2020). The following different etiologies have been suggested for this type.

##### Deficit in IPS

Various authors suggested that the deficit principally arises from an impairment of magnitude-related circuits in the IPS itself (Chan and Wong 2020; Skagerlund and Träff 2016; Wilson and Dehaene 2007), making the IPS as the number sense module of the triple-code model the first dysfunctional instance even before formal learning of arithmetic (for reviews, see Siemann and Petermann 2018; Von Aster and Shalev 2007). The affected individuals have an impairment in the acuity of the ANS (Landerl et al. 2009; Mazzocco et al. 2011; Piazza et al. 2010), which affects their ability to estimate the quantity of and discriminate between sets of items. It also leads to an impaired association of numerals with the underlying quantity information and hence affects the understanding of number symbols (i.e., Arabic numbers). In turn, this compromises the acquisition and consolidation of adequate basic arithmetic skills such as multi-digit calculation and arithmetic fact retrieval (Dehaene 1992; Feigenson et al. 2004; Piazza 2010).

This idea would be well in line with the two-network framework under the premise that (a) the IPS is affected bilaterally and (b) the IPS is the sole region mediating the encoding of numerical magnitude information. However, recent evidence from high-field fMRI studies revealed areas that are tuned to numerosity not only in parietal cortex (Harvey et al. 2013) but also in frontal and inferior temporal regions (Harvey and Dumoulin 2017). This has been interpreted as evidence for a distributed representation of numerical magnitude information that informs multiple cognitive systems such as decision making, attentional control or motion perception. Alternatively, the observed numerosity maps may emerge by receiving major input from the crucial node in IPS. If not only the IPS is affected but also its dorsal connectivity (Fig. 1), then such a defective ANS hypothesis would be well in line with the two-network model.

##### Deficit in the visual dorsal processing pathway

Chan and Wong (2020) describe a group of children that showed deficits in numerosity processing but did not seem to have significant difficulties in basic arithmetic. The authors called this group “The numerosity coding deficit group” and did not yet propose a neural underpinning. Against the background of the two-network framework (see also Roggeman et al. 2011; Santens et al. 2010), we would suggest that a functional and/or structural deficit in the visual dorsal processing pathway connecting visual occipital cortex with the IPS might underlie this subgroup. In this case, the non-symbolic numerosity input would be already impaired before entering the IPS, while symbolic IPS processing would not be affected. This would imply, however, that functioning non-symbolic numerosity processing is not a necessary prerequisite for the acquisition of symbolic processing skills. It remains debated whether or not the acquisition of symbolic number knowledge requires the association of non-symbolic number information with the corresponding symbol (De Smedt et al. 2013; LeFevre et al. 2022).

#### Number-unspecific subtypes

##### Working memory subtype

Various studies have shown a DD subtype which is associated with working memory deficits (V. Menon 2016; Szucs et al. 2013). Dependent on the authors, this group is referred to as “Working Memory Deficit Group” (Chan and Wong 2020) with primarily visuospatial working memory deficits or as “Magnitude Processing and Domain-General Subtype” (Träff et al. 2017) with a non-symbolic and symbolic number processing and enumeration deficit with a combined visuospatial working memory deficit, but intact subitizing ability, or as “Verbal Working Memory Deficit group” (Landerl et al. 2017). Against the background of the TNF, deficits in frontal cortex as well as its connections with the parietal cortex should be prevailing in all these groups. Crucially, the dorsal pathways connecting frontal and parietal cortex (superior longitudinal fascicle II) mature later (around the 7th year of life) than the ventral pathways in middle and late childhood (Friederici 2012); therefore, a working memory subtype dyscalculia that involves impairments of dorsal pathways should manifest later in development than those that involve gray matter areas (e.g., frontal cortex) only.

##### Motoric subtype

Landerl et al. (2017) define a motor subtype of dyscalculia that is associated with a deficiency in finger arithmetic, as assistance with procedure arithmetic can be helpful, particularly during development. Based on the two-network framework, we would not suggest this as a separate subtype of DD, but suggest that if there is another deficit that leads to DD in the size processing or fact network, it may actually be strongly disadvantageous if then, e.g., in addition to a deficit in the IPS or in the core fact network of AG, supramarginal gyrus, superior temporal gyrus and thalamus, finger representation cannot be used to help. This was shown in adult dyscalculia patients who take the fingers to help with a fact retrieval problem (Göbel et al. 2022; Kaufmann et al. 2011a, b) and in acalculia patients with multiplication deficits (Smaczny et al. 2021). In the two-network framework, this is represented by the connection of thalamus and primary motor cortex area, which have also been shown to host numerosity tuned number maps (Harvey and Dumoulin 2017; see also Ardila (2020) for a review on cortico-subcortical lesions as one explanation for finger agnosia in Gerstmann’s syndrome).

### Impairments in both networks

The TNF proposes that the left visual number form area is providing input to both networks (i.e., the magnitude network and the fact retrieval network). Against this background, we hypothesize that a deficit in the left visual number form area itself should have a negative effect on the association of symbol number magnitude, which should affect both magnitude processing and calculation as well as arithmetic fact retrieval. We suggest, however, that during development this deficit should become apparent first in magnitude processing, estimation and calculation, before later also the ability to build and retrieve arithmetic facts in memory will be affected. Due to the vicinity of the VNF and the left-lateralized visual word form area, we would hypothesize that these children would be probably prone to deficits in processing other written symbols as well, which might further contribute to the higher than expected comorbidity between dyscalculia and dyslexia (Moll et al. 2019; Thapar et al. 2017). However, so far there has not been reported evidence of more dyscalculia in visual subtypes of dyslexia.

### Impairments in network interaction

The interaction between magnitude processing and fact retrieval is likely to be mediated not only via U-fibers between IPS and AG (Klein et al. 2016), but also via the triangle AG—thalamus—IPS as specified above (Fig. 1). This leads to the following possible impairments:

An impairment of the *U-fibers between AG and IPS* is likely to lead relatively specifically to impairments in the interaction between the two networks of magnitude processing and fact retrieval. This might be evident only in higher reaction time data, because IPS and AG are connected polysynaptic via the thalamus as well. Possible comorbidities of this impairment should affect relatively specifically associative learning processes as the region between angular gyrus and IPS has been shown to be involved in associative learning and memory-guided behavior in general rather than being specifically devoted to mathematical content (Grabner et al. 2009a, b; Grabner et al. 2009a, b; Ranganath and Ritchey 2012).

Similarly, an isolated impairment of the structural connectivity between *thalamus and IPS* or between *thalamus and AG* should also moderately impair the interaction between magnitude and fact retrieval network. An impairment of this connection, however, might also partially affect further domain-general functions subserved by AG and thalamus, which typically serve as cross-modal hub such as, for instance, word reading and comprehension or reorienting attention to relevant information (for a review see Seghier 2013).

The data by Smaczny et al. (2021) suggest that an isolated deficit in the left IPS should also lead to impairments in the interaction between the two networks rather than the inability to manipulate magnitudes. In particular, lesions leading to a disconnection of the left IPS resulted in the inability to retrieve arithmetic facts from memory, while addition, subtraction or magnitude comparison abilities were spared. Since the TNF suggests that magnitude manipulations are redundantly performed in the bilateral IPS, we hypothesize that magnitude processing and calculation in the presence of an acquired or developmental deficit in the left IPS may be compensated for by the contralateral IPS. However, the shift from more effortful procedural calculation (subserved by the magnitude network) to arithmetic fact retrieval (subserved by the retrieval network) as it typically occurs during arithmetic learning might be hindered as this is tied to the left IPS. This should not only result in the use of effortful arithmetic procedures where fact retrieval would be highly beneficial (e.g., 3 + 3 + 3 + 3 instead of 4*3) but also affect the performance of procedural calculation itself (e.g., subtraction) as the two-network framework posits that also within more difficult subtraction tasks fact retrieval will be used (e.g., when decomposing the calculation into easier subcomponents such as 37–2 = (7–2) + 30). Similar cases have recently been described for dyscalculia in adulthood (Göbel et al. 2022; Kaufmann et al. 2011a, b). Notably, processes that are preferentially processed in the left IPS might be affected as well (e.g., parity judgment, (Klein et al. 2010)). Importantly, however, due to the basically redundant arrangement of size processing in the IPS of both hemispheres, no or minimal impairments should develop for the approximate number system, magnitude comparison of quantities or symbolic numbers, or in arithmetic (addition, subtraction). Thus, this disturbed interaction between networks should eventually result in a clinical picture similar to that of arithmetic fact dyscalculia.

When there is an isolated problem of the left AG, switching between the networks should be impaired and so should be arithmetic fact retrieval (similar to the IPS deficit). However, task switching would be no longer in the foreground, because it would lead to various mathematical (e.g., deficits in arithmetic fact acquisition and retrieval) and non-mathematical impairments (deficits in written language acquisition).

Finally, a problem within the thalamus (e.g., reduced gray matter) is also likely to have far-reaching mathematical and non-mathematical cognitive consequences.

## The TNF against the background of recently published studies of DD in children

The current proposal is meant to stimulate, inspire and structure future research in the domain of developmental dyscalculia. Nevertheless, any new theoretical proposal needs to demonstrate that it can explain existent empirical data. In the case of the TNF, this fundamental requirement is complicated, however, by a number of factors. First, scrutinizing whether a given study is in line with the predictions of the TNF requires to categorize the tasks and stimuli used as tapping primarily into one of the two networks proposed here. While this seems rather obvious for healthy adults, it is far less obvious for patients or children suffering from DD. For example, tasks such as simple addition (e.g., 2 + 3 = ?) may well be classified as mainly relying on the fact retrieval network for healthy adults. Yet, for children suffering from DD, this task may represent a major challenge and solving may involve a multitude of compensatory strategies (e.g., counting all using 5 fingers on two hands) to solve. Similarly, a task that is classified as pure magnitude processing such as the number magnitude comparison task may be solved by alternative solutions in children suffering from DD, such as counting until arriving at the involved numbers, for example. These difficulties may then lead to a lack of hallmark effects such as the numerical distance effect in a magnitude comparison task in children suffering from DD (Ashkenazi et al. 2012; Mussolin et al. 2010). A second problem is that many (in particular older) studies focus on univariate analyses that do not allow the analysis of the underlying activation patterns and hence do not allow to adjudicate whether a given activity in the IPS in one task context (e.g., fact retrieval) is comparable to the pattern in another context (e.g., magnitude processing). This renders interpretation difficult and leaves room for reverse inference. Furthermore, many studies do not analyze the functional or structural connectivity and hence remain mute to the network characteristics that are central for the TNF.

A formal review of all neuroimaging studies on DD is beyond the scope of this contribution. Nevertheless, we briefly scrutinize whether the available data allow to test the core prediction of the TNF.

A number of studies have compared univariate brain activity in children suffering from DD with typically developing (TD) children using symbolic (Mussolin et al. 2010; Üstün et al. 2021) and/or non-symbolic magnitude comparison tasks (Kucian et al. 2011; Üstün et al. 2021), numerical ordering tasks (McCaskey et al. 2018), complex (Ashkenazi et al. 2012) or simple addition (Rosenberg‐Lee et al. 2015), subtraction (Peters and De Smedt 2018; Rosenberg‐Lee et al. 2015) or multiplication problems (Berteletti et al. 2014). Crucially, these studies report either more (Peters and De Smedt 2018; Rosenberg‐Lee et al. 2015) or less (Berteletti et al. 2014) parietal activity when comparing DD with TD children, depending on the task. This underlines the complex role of the IPS across tasks and casts some doubt on the conclusions that we can draw from these findings. Moreover, the IPS is involved in both networks in the TNF. Although the TNF proposes a similar function for the IPS within both networks (i.e., subserving magnitude processing), this renders it a bad candidate for formulating clear-cut hypothesis.

On a more general note, the interpretation of univariate amplitude differences between TD and DD children is ambivalent because since they can either be interpreted either in terms of lack of involvement of a function in a given task context (hypoactivation) or a compensatory activation of additional resources (hyperactivation).

More informative, several studies analyze group differences in terms of a given functional index such as the distance effect, for example. Mussolin et al. (2010) and Ashkenazi et al. (2012) report that children suffering from DD failed to show a typical modulation of parietal activity as a function of numerical distance. This can be interpreted as evidence for an atypical and dysfunctional recruitment of parietal areas in number magnitude processing in DD children – in line with the involvement of the IPS in the magnitude network.

Several studies investigated effective connectivity in simple addition and subtraction tasks (Rosenberg‐Lee et al. 2015) or ordering tasks (“Judge of the triplet 3__6__5 is in ascending order!”; Michels et al. 2018). Both studies analyze effective connectivity based on a parietal seed region and report that DD children exhibit hyperconnectivity with a number of areas in parietal, prefrontal and anterior cingulate cortex. A number of these sites belong to either of both networks proposed by the TNF. Although children in Rosenberg-Lee and colleagues (2015) were particularly impaired in subtraction problems that are thought to primarily tap into the magnitude processing network, aberrant functional connectivity was largely driven by addition which is tapping into both magnitude processing and fact retrieval networks. Since DD children were comparable accurate but significantly slower, we can hypothesize that the aberrant connectivity reflects compensatory activation of both networks in the context of addition problems. The areas showing hyperconnectivity with the IPS do indeed show considerably overlap with both networks proposed by the TNF. Hence, the results can tentatively be interpreted along the lines of the TNF. Yet, to probe the functional relevance of the nodes proposed by the TNS, effective connectivity with different seed regions would be informative.

In sum, the current proposal remains speculative for the moment. Probing the functional relevance of the nodes and their contribution to the proposed networks remains challenging on the basis of the scarce data basis. More multi-center studies with a joint set of magnitude- and fact retrieval-related tasks are required.

In their recent meta-analysis, Menon et al. (2020) adopt a systems neuroscience approach and integrate the results of existing, mostly univariate functional neuroimaging studies that have primarily focused on the IPS as the locus of numerical information processing deficits in dyscalculic children with findings on domain-general factors. The authors suggest that dyscalculia arises from a complex interplay between “domain-specific” (i.e., quantity processing and arithmetic fact retrieval/problem-solving) and “domain-general” deficits (mostly visuospatial working memory and cognitive control) that are implemented in overlapping brain circuits. In particular, impairments in one or more of a inter-connected set of brain regions that include the IPS and other parietal cortex regions, inferior temporal cortex, medial temporal lobe, and multiple PFC areas are supposed to compromise the efficiency of numerical problem-solving skills and constitute risk factors for dyscalculia.

The TNF adopts a similar approach in that we too believe that a systems neuroscience approach is necessary for understanding dyscalculia. The focus is no longer on isolated dysfunctional areas per se, but on their complex interaction in the context of distributed but connected functional systems. Our proposal differs in so far as the TNF assumes that each of two domain-specific aspects of numerical cognition (magnitude vs. facts) is processed by a separate network that overlaps with areas involved in domain-general processes (e.g., working memory). The fundamentally different assumption about the composition of the networks distinguishes the TNF from the approaches put forward by Menon et al. (2020) and lead to diverging hypotheses concerning possible causes and associations in dyscalculia. Both approaches do, however, jointly emphasize the importance of considering the integrity of overlapping functional networks.

### Limitations and perspectives

Just like the triple-code model, the TNF is primarily based on findings from educated adults with matured neuronal circuits and arithmetic expertise. The model, therefore, does not take into consideration the nonlinear developmental trajectories of mathematical learning. However, children differ from adults with respect to brain maturation and synaptogenesis (see Ardila and Rosselli 2002) and show a transient contribution of hippocampal areas, for example (Iuculano et al. 2015). Thus, it has to be noted that the TNF is not a model of mathematical learning. A further limitation of the current proposal is that the data on developmental dyscalculia is still limited. Overall, there are few studies on the domain of developmental dyscalculia and of these, several studies are underpowered. The scarce data basis makes it difficult to draw profound conclusions about the phenomenological subtypes and their underlying neural origin in developmental dyscalculia. Here, the present proposal is intended to serve as a stimulus for future research in the domain of developmental dyscalculia.

## Conclusion

We specified and amended the two-network framework (TNF) of numerical cognition in adults with recent results from disconnectome mapping. Crucially, we suggest that an impairment within the two different networks and/or their interaction results in specific and identifiable deficit patterns. We assume that these main characteristics should already emerge earlier during development because the model represents the endpoint of the acquisition and use of mathematical competencies in adults. Thus, in line with these main characteristics of the TNF, we propose four major subtypes of DD. These different profiles have previously been accounted for by specific and isolated mechanisms with hypothetical neural underpinnings that did not stand up to empirical scrutiny. The TNF provides a more coherent framework with testable predictions.

The TNF had its starting point in connectivity and functional MRI data collected in healthy adults. With data coming from acalculic and dyscalculic adults, the model was updated and proved suited to explain the observed deficits. Here, we deduce four major profiles of DD and compare them against previously proposed taxonomies of DD. Most importantly, as opposed to previous (often mono-causal) models of the neural origins of DD, the TNF provides a coherent framework that has evolved from empirical observations. The four major subtypes of DD proposed here reflect the observed diversity of symptoms. The strength of the current proposition is that it can integrate specific hypotheses about circumscribed subtypes (e.g., sensitivity interference) and provide a meaningful and straight-forward neural implementation of DD subtypes that have been derived from cognitive profiles.

The current proposition is not the first model that takes into account the idea that numerical cognition (much like many other cognitive functions) relies on a network of distributed nodes with specific functions. Amalric and Dehaene (2019) proposed that mathematical knowledge forms a dedicated area of semantic knowledge with a distinct cortical network comprising bilateral parietal and temporal areas. Notably, Amalric and Dehaene (2019) did not observe a contribution of our arithmetic facts network with nodes around the angular gyrus. This, however, may have been due to the rather complex nature of the items which may lean towards the right pole of the proposed complexity gradient with a prevailing contribution of the magnitude network. In addition, it should be noted that the two-network framework makes more specific predictions concerning both gray and white matter contributions to numerical cognition compared to the network proposed by Amalric and Dehaene (2019). Iuculano and Menon (2018) propose that numerical cognition is based on “neural building blocks […] that are constructed from core hubs anchored in the IPS and the FG and their associated functional circuits.” Taking into account the complexity of the involved networks and their developmental trajectories, the authors conclude that in order to understand the neural origins of DD, we need to adopt “a systems neuroscience approach, with its emphasis on networks and connectivity, rather than a pure localization approach.”

The current proposition adheres to this general approach and converges on the idea that the diverse cognitive phenotypes of DD cannot be accounted for by mono-site, mono-causal models. The current model should be understood as a starting point for a more integrative, systems neuroscience approach to DD. Most crucially, this would also require to move from single-laboratory, small-scale projects to multi-center, large-scale projects, involving researchers from various academic disciplines such as cognitive psychology, cognitive neuroscience and medicine, as well as practitioners.

## Data Availability

This opinion article is based on literature review. Thus, no data was acquired or can be made available.
